# Influence of micro- and nano-bubble treatment on morphological characteristics and flow properties of spray-dried milk protein concentrate powders

**DOI:** 10.3168/jdsc.2022-0226

**Published:** 2022-09-03

**Authors:** K.S. Babu, K. Siliveru, J.K. Amamcharla

**Affiliations:** 1 Department of Animal Sciences and Industry, Food Science Institute, Kansas State University, Manhattan 66506; 2 Department of Grain Science and Industry, Kansas State University, Manhattan 66506

## Abstract

This study investigated the morphological and bulk handling properties of milk protein concentrate (MPC) powders manufactured from incorporating micro- and nano-bubbles (MNB) before spray drying. Control MPC powders (C-MPC; no MNB treatment) and MNB-treated MPC powders (MNB-MPC; MPC dispersions passed through the MNB system and subsequently spray dried) were characterized in terms of particle size, shape factors, stability, variable flow rate, shear cell tests, compressibility, and wall friction. The MPC powders produced after the MNB injection process had better flowability and lower basic flow energy. Shear tests showed that C-MPC powders were more cohesive than MNB-MPC powders. The MNB-MPC powders had lower flow rate index values, lower wall friction angles, more rounded shape, and significant differences in powder compressibility compared with C-MPC powders. Overall, the results demonstrated that MNB incorporation during spray drying can produce ingredients with comparable morphological characteristics while improving the bulk powder properties.

## Highlights

• Treatment with MNB was used to improve MPC powder flowability.
• Basic flowability energy values of MPC powders decreased with MNB injection.
• Control MPC powders were more cohesive than MNB-MPC powders.

## Graphical Abstract

**Figure:**
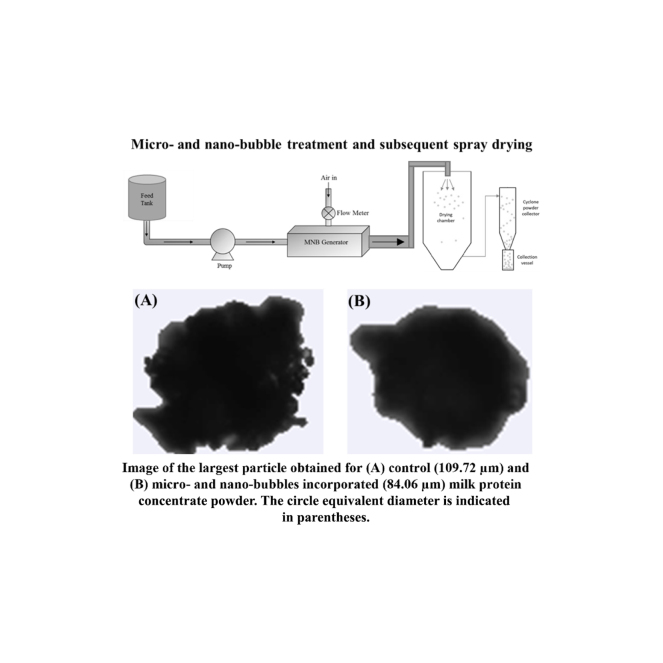
**Summary:** Milk protein concentrate (MPC) is an ideal dairy ingredient to provide nutritional and functional benefits in highprotein dairy and food products. However, it is also a challenge for the end-user to incorporate MPC powders in a formulation due to their deprived bulk handling properties. Therefore, simple and innovative strategies to improving final physical powder properties are of critical consideration for the dairy industry. In the present work, we are exploring the possibility of utilizing micro- and nano-bubbles for improving morphological characteristics and flow properties of spray-dried MPC powders. Micro- and nano-bubble (MNB) incorporation exhibited lower particle size and had better flowability. The differences in particle structure and shape appear to have a large impact on its physical characteristics and will likely also influence the rehydration properties.

The high-protein, low-lactose content with attractive functional and nutritional qualities make milk protein concentrate (**MPC**) powders an ideal ingredient in various dairy and food product formulations. However, impaired rehydration and bulk flow characteristics during storage make it challenging for end-users to incorporate MPC powders into a formulation. Protein content and storage time and temperature are some of the critical factors that influence the functionality of MPC powders (Babu and Amamcharla, 2018, 2021). The flow properties of skim milk powder, whole milk powder, whey protein concentrates, and MPC powders have been well characterized (Kim et al., 2005; Fitzpatrick, 2007; Crowley et al., 2014). Modifying powder particle structure may influence the physical attributes of the powder (density, particle size, porosity, and particle morphology), and these influence the bulk handling of powders industrially. Bouvier et al. (2013) used extrusion-porosification to produce MPC powders with improved dispersibility and increased particle porosity. After spray drying, the powder is transferred to storage bins or silos and can go through numerous handling steps (Ilari, 2002). Additionally, as a dairy ingredient, MPC powders are likely to be stored in silos before usage. Therefore, understanding the overall flow behavior and how they may behave during storage and handling is critical. The MPC35 are considered to be free-flowing, whereas MPC with 60% and 70% were easy flowing (Crowley et al., 2014), and MPC80, MPC85, and MPC90 were noted to be cohesive and were claimed to have relatively poor flowability (Crowley et al., 2014; Babu et al., 2018). Therefore, alternative techniques to improve flow characteristics of MPC are of significant interest.

Microbubbles (**MB**; size range: 10–50 µm) and nano-bubbles (size: <200 nm) are gas-filled entities within a bulk liquid (Agarwal et al., 2011; Temesgen et al., 2017). The techniques to incorporate micro- and nano-bubbles (**MNB**) in bulk liquid include hydrodynamic and acoustic cavitation, electrochemical cavitation, membranes, and mechanical agitation (Thi Phan et al., 2020). Micro- and nano-bubbles offer substantial improvements in applications such as wastewater treatment, mining, agriculture, food processing, and medicine (Liu et al., 2008; Amamcharla et al., 2017; Temesgen et al., 2017; Jannesari et al., 2020). Recently, the potential applications of nano-bubbles in food and dairy processing industries have been thoroughly reviewed (Thi Phan et al., 2020; Babu and Amamcharla, 2022b; Zhang et al., 2022). Ebina et al. (2013) noted a significant increase in O_2_ content in water (7.7 to 31.7 mg/L) when incorporated as O_2_ MNB. In the food processing sector, the use of CO_2_ and N_2_ is getting more attention. This could be due to their high solubility and relatively low reactivity. Application of CO_2_-MNB significantly decreased the scooping hardness and melting rate of soft-serve ice cream, and also enhanced the overall acceptability of soft-serve ice cream (Adhikari et al., 2020). Very recently, Babu et al. (2022) envisaged MNB treatment as a new process method to improve the rheological and functional characteristics of Greek-style yogurt. Previously, McSweeney et al. (2021) observed better rehydration behavior of N_2_ injected MPC powders. The influence of CO_2_ injection on the physical and functional properties of whole milk powder has been described by Kosasih et al. (2016). They noted that CO_2_ injection before spray drying increased powder dispersibility, porosity, and occluded air content. Hanrahan et al. (1962) examined the impact of N_2_ injection into whole-milk concentrate before spray drying and described an increase in dispersibility and powder particle size. However, limited research has been implemented regarding the use of gas injection to modify powder particle structure and improve the subsequent flow properties of high-protein dairy ingredients. The effect of MNB on the physical properties of high-protein powders has not been previously investigated but may assist the manufacture of MPC powders with improved flow behavior. Therefore, the objective of this research was to characterize the physical and bulk handling properties of MNB-MPC powders compared with control MPC.

A benchtop MNB generation system based on hydrodynamic cavitation was designed and assembled at Kansas State University. More details about the MNB treatment procedure can be found elsewhere (Babu and Amamcharla, 2022a). The MPC dispersions were prepared using 2 independent lots of MPC powder with 85% protein content obtained from a commercial dairy ingredient provider within the United States. The average protein, moisture, fat, lactose, and ash contents as per the certificate of analysis provided by the supplier for the MPC85 were 86.85% (wt/wt), 5.25% (wt/wt), 0.93% (wt/wt), 4.79% (wt/wt), and 6.68% (wt/wt), respectively. Control MPC (**C-MPC**; MPC dispersions pumped through the diaphragm pump without air injection) and MNB incorporated MPC (**MNB-MPC**; MNB treated using a venturi injector; Hydra-Flex) were prepared and subsequently spray-dried in a laboratory-scale spray dryer (YC-015, Shanghai Pilotech Instrument and Equipment Co. Ltd.). The inlet temperature was set at 180°C, and the outlet temperature ranged between 60 and 65°C. The spray pressure was maintained at 206.84 kPa, and the average relative humidity was 52%. The spray-dried MPC powders were collected and sealed in Whirl-Pak bags (Nasco). Subsequently, the morphological and flow characterizations of the C-MPC and MNB-MPC powders were performed in duplicate. The morphological and flow characteristics of the resultant MPC powders were analyzed using PROC GLMMIX procedure of SAS (version 9.4, SAS Institute Inc.).

Morphological characteristics of C-MPC and MNB-MPC powders were analyzed by a Malvern Morphologi G3ID (Malvern Instruments) following the method by Babu et al. (2018). The circle equivalent diameter (**CED**), high sensitivity circularity (**HSC**), elongation, solidity mean, and convexity were calculated from the 2-dimensional images. Circularity (range 0 to 1) describes how close the shape of the particle is to a perfect circle, whereas convexity (range 0 to 1) is a measure of the surface roughness of a particle. A smooth particle has a convexity of 1, whereas an irregularly shaped particle or a very spiky one has a convexity closer to 0. Circle or square has an elongation value of 0, whereas shapes with large aspect ratios have an elongation closer to 1. Solidity describes the amount to which a shape is convex or concave. The solidity of a completely convex shape is 1, whereas the farther the solidity deviates from 1, the greater the extent of concavity in the structure.

The FT4 powder rheometer (Freemans Technology) was used to evaluate the powder rheology of the C-MPC and MNB-MPC powders following the method described by Babu et al. (2018). The samples were conditioned using the conditioning cycle to ensure the repeatability of the results and remove any packing stress. Measuring the flow properties for characterizing powder flow using FT4 powder rheometer include various flow, compressibility, and shear cell tests. Basic flowability energy (**BFE**), stability index (**SI**), specific energy, and flow rate index (**FRI**) were obtained from the flow tests. The measurements were performed using the cylindrical cell in which powders were pre-consolidated under 9 kPa of normal stress. Cohesion, unconfined yield strength (**UYS**), major principal stress (**MPS**), and flow function (**FF**) were obtained from the shear tests. Bulk density was measured by weighing 2 g of the C-MPC and MNB-MPC powder and placing it into a 50-mL graduated cylinder. The loose/poured powder bulk density is the ratio of the mass of powder (g) and the volume (mL) occupied in a graduated cylinder (Tonon et al., 2011).

The morphological properties of C-MPC and MNB-MPC powders are summarized in Table 1. The CED of MPC powder particles decreased with the MNB treatment. Particle size of the final powder is dependent on the viscosity of the feed (Caric and Kalab, 1987). Keogh et al. (2003) also found that the particle size of spray-dried milk powders increased linearly with an increase in the apparent viscosity. The HSC results confirmed that the morphology of MNB-MPC powder exhibited more round-shaped particles compared with C-MPC powders (Table 1). Interestingly, the aspect ratio, elongation, solidity, and convexity were comparable for both the C-MPC and MNB-MPC powders. Similarly, Kudo et al. (2020) studied the effect of particle size distribution on flowability of granulated lactose and noted dependency of flow properties on particle shape was minimal. Figure 1 shows the 2-dimensional shape of the largest C-MPC and MNB-MPC powder particles. The measured CED was 7.24 μm for the C-MNC. In contrast, CED was significantly (*P* > 0.05) lower with the MNB treatment and was found to be 6.91 μm for the MNB-MPC powder particles. The particle size changes were identical to those previously noted for hydrodynamic-cavitated MPC powders (Li et al., 2018). In contrast, the incorporation of N_2_ into the MPC concentrate significantly increased the size of the regular MPC powder particles from 73 to 78 μm. However, the CO_2_ injection of whole milk concentrates showed a particle size ∼15 μm (D50) for both the control and treated powder samples. Milk concentrates with a higher viscosity form larger droplets when atomized, resulting in powder with a larger particle size (Anandharamakrishnan, 2017; Power et al., 2020). The observed differences in particle size, structure, and shape appeared to have a large impact on its physical characteristics and have also positively influenced the rehydration behavior (Babu and Amamcharla, 2022a). Several powder properties such as solubility, morphology, and flow characteristics are determined by its particle size distribution, which is primarily governed by feed characteristics and conditions maintained during further processing of the retentates (Schuck, 2011). Overall, morphological results suggested that MNB treatment before spray drying would not drastically alter the final powder morphology while delivering desired viscosity reductions, helping in potential energy savings in the spray-drying process.

**Table 1:** Morphological characteristics, dynamic flow, and shear flow properties of control (C-MPC) and micro- and nano-bubble-treated milk protein concentrate (MNB-MPC) powders

Property	C-MPC	MNB-MPC
Circle equivalent diameter (μm)	7.27 ± 0.05^a^	6.91 ± 0.01^b^
High sensitivity circularity	0.883 ± 0.001^a^	0.893 ± 0.001^b^
Aspect ratio	0.824 ± 0.000^a^	0.823 ± 0.000^b^
Elongation	0.175 ± 0.000^a^	0.177 ± 0.000^b^
Solidity	0.981 ± 0.000^a^	0.979 ± 0.000^b^
Convexity	0.989 ± 0.000^a^	0.986 ± 0.000^b^
Basic flow energy (mJ)	507.59 ± 6.39^a^	434.05 ± 6.85^b^
Stability index	1.03 ± 0.00^a^	0.96 ± 0.02^b^
Flow rate index	1.58 ± 0.03^a^	1.42 ± 0.04^a^
Specific energy (mJ/g)	40.66 ± 1.97^a^	17.43 ± 0.49^b^
Unconfined yield stress (kPa)	6.13 ± 0.02^a^	3.37 ± 0.06^b^
Cohesion (kPa)	1.60 ± 0.01^a^	1.02 ± 0.01^b^
Major principle stress (kPa)	15.82 ± 0.22^a^	12.29 ± 0.07^b^
Angle of internal friction (°)	35.89 ± 0.80^a^	27.26 ± 0.97^b^
Flow function coefficient	2.58 ± 0.03^a^	3.50 ± 0.11^b^

a,b Mean values for different shape factors and flow or shear properties within a row with different superscripts differ (*P* < 0.05); n = 4.

**Figure 1:**
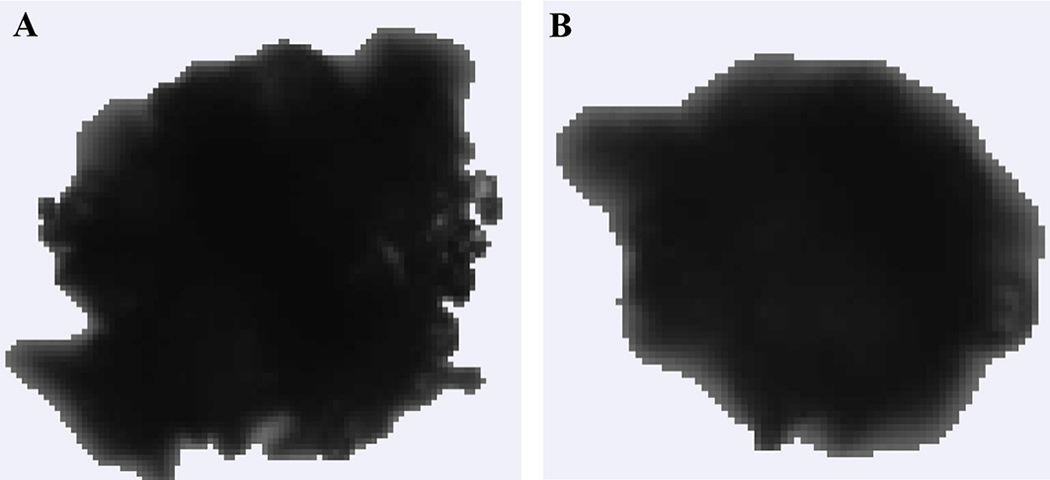
Two-dimensional images showing the shape obtained from the Morphologi G3ID (Malvern Instruments) for the largest particle observed for the (A) control and (B) micro- and nano-bubble-treated milk protein concentrate powders.

The dynamic flow and shear flow properties of C-MPC and MNB-MPC powders are summarized in Table 1. The BFE value of C-MPC was significantly different (*P* < 0.05) from that of MNB-MPC powder, indicating that the flow behavior of MNB-MPC powder would be better than the C-MPC powder (Table 1). It can be noted that BFE of MNB-MPC powders was relatively lower compared with C-MPC powder samples composed of smaller or irregular particles with greater interparticle friction. Previously, Lapčík et al. (2015) reported lower BFE values (127 to 157 mJ) for skim milk powder, demineralized whey powder, and whey powders. However, the BFE of MPC powders generally ranges from 510 to 930 mJ (Babu et al., 2018). The C-MPC and MNB-MPC powder are relatively stable and do not agglomerate during handling after being subjected to flow under high stress. The SI of C-MPC and MNB-MPC powders were 1.03 and 0.96, respectively, and can be considered acceptable for a stable powder (0.9 to 1.1); more than 1.1 would be considered unstable (Bian et al., 2015). Both C-MPC and MNB-MPC powder had average flow rate sensitivities because the FRI value was less than 1.5. This shows that the C-MPC and MNB-MPC powders were not remarkably cohesive (Leturia et al., 2014). Indeed, the FRI of C-MPC was not significantly (*P* > 0.05) different from the MNB-MPC powders. The specific energy decreased significantly (*P* < 0.05) with the MNB treatment. The specific energy values depend on the cohesive and mechanical interlocking forces between the powder particles and are only slightly influenced by compressibility. The specific energy value for MNB-MPC was lower than C-MPC, indicating that it was the least cohesive sample in an unconfined flow regimen and was consistent with a relatively low BFE value. Overall, a higher BFE value (lower flowability) and higher specific energy value (higher cohesivity) of C-MPC as compared with MNB-MPC showed that it was a less stable sample. The general trend observed was an improvement of the dispersion, flow, and packing properties after the MNB treatment. The difference in flow energies between the C-MPC and MNB-MPC powders can be explained by the differences in size distribution, shapes, and bulk density (0.187 ± 0.006 and 0.151 ± 0.003 g/mL for C-MPC and MNB-MPC powders, respectively). The C-MPC exhibited higher flow energy than the MNB-MPC, which is attributed to particle mechanical interlocking resulting from the higher surface roughness and dents, revealed from the scanning electron microscopy images (Babu and Amamcharla, 2022a). It was previously found by Fu et al. (2012) that powders with the highest sphericity had better flowability. Similarly, in this study, MNB-MPC had the most spherical powder particles (Table 1).

Shear properties provide insights into whether the C-MPC and MNB-MPC powders will move from a static condition to dynamic flow or whether bridging, blockages, and stoppages are likely. The UYS, cohesion, MPS, and AIF of C-MPC powders were higher than MNB-MPC powders, indicating that under consolidation, the flow behavior of MNB-MPC would be better than that of C-MPC powders. The higher cohesion and AIF values of C-MPC powders suggest that there could be potential interlocking. The UYS decreased from 6.13 to 3.37 kPa with the MNB treatment. The shear parameters AIF and MPS significantly (*P* < 0.05) decreased from 35.89 to 27.26° and 15.82 to 12.29 kPa, respectively, with the MNB treatment. Likewise, Crowley et al. (2014) reported a wall friction angle of 21.7° for a regular MPC80 powder, which differs from the value for C-MPC powders in the current study, possibly due to differences in powder particle size reported in the current study. Based on the FF classification by Jenike (1976), a powder is cohesive if its FF ranges between 2 and 4 and very cohesive between 1 and 2. Therefore, the flow behavior of C-MPC and MNB-MPC powders could be classified as cohesive, although the MNB-MPC had a significantly (*P* < 0.05) higher FF value. The higher the FF value, the easier the bulk solid can flow (Bian et al., 2015). The differences in the flow properties of powder particles can be due to interparticle interactions during flow (Mitra et al., 2017). Rennie et al. (1999) noted that powder cohesiveness decreases as particle size increases. However, both C-MPC and MNB-MPC powders were classified as cohesive despite having a higher CED for C-MPC powders. Correlating SEM micrographs (Babu and Amamcharla, 2022a) and shear data, it was evident that C-MPC powders encouraged higher particle-to-particle interaction than MNB-MPC powders.

Furthermore, the results from compressibility testing revealed that the percent change in volume increased with normal stress applied for both C-MPC and MNB-MPC powders (Figure 2). The rearrangement of finer particles in the MNB-MPC could have resulted in higher compressibility. This could be due to the compact particle packing and increases in interparticle surface contact. Previously, McSweeney et al. (2021) noted that N_2_ injected MPC powders were more compressible (50.4%) compared with regular MPC powders (41.5%). Likewise, fine particles exhibit higher compressibility than the coarser ones because of the greater surface area (Jan et al., 2017). Overall, the morphological and flow results suggested that MNB-MPC powders had a higher proportion of regular-shaped particles and a comparatively smoother surface (Table 1), confirming that it was more easy flowing or less cohesive than C-MPC powders. These observed differences in particle structure and shape also play a critical role in the physical characteristics and rehydration properties of MPC powders (McSweeney et al., 2021; Babu and Amamcharla, 2022a).

**Figure 2:**
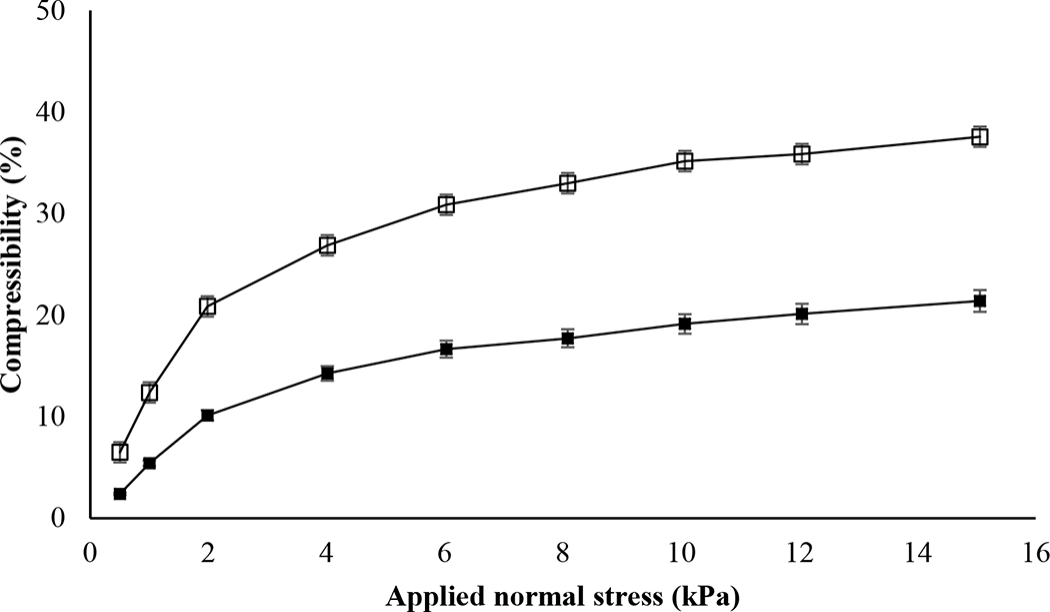
Compressibility of the control (▪) and micro- and nano-bubble-treated (□) milk protein concentrate powders; n = 4.

Technologies with MNB have received substantial attention for industrial applications due to their low cost, eco-friendliness, and scale-up ease. MNB treatment is a robust process technology and MNB treatment resulted in improvement of flow performance of MPC powders. The findings of this study indicate that MNB treatment can be used as a physical pre-treatment before spray drying, and it represents potential for a more energy-saving drying process while providing better powder flow properties without having an impact on morphological characteristics.
